# 4545 Identifying Symptom Pattern Trajectories among Heart Failure Patients in a Palliative Care Trial: A Work In Progress

**DOI:** 10.1017/cts.2020.400

**Published:** 2020-07-29

**Authors:** Macy Lynn Stockdill, Christopher Lee, J. Nicholas Dionne-Odom, Bradley Aouizerat, Raegan Durant, Andres Azuero, Marie Bakitas

**Affiliations:** 1 University of Alabama at Birmingham; 2 Boston College; 3 New York University

## Abstract

OBJECTIVES/GOALS: This work-in-progress aims to: 1) identify and differentiate symptom pattern trajectories in a sample of older adult heart failure (HF) patients over 24 weeks, and 2) examine associations between sociodemographic/clinical/physiological characteristics, dyadic health, and symptom trajectories. METHODS/STUDY POPULATION: ENABLE CHF-PC, a palliative care RCT (NCT02505425), was conducted at a Southeastern US medical center. Between 2016-2018, 415 older adult HF patients and 159 family caregivers were randomized to receive a psychoeducational intervention or usual care. Baseline sociodemographic information (age, gender, rurality, etc.) were collected. Outcome variables of interest include symptoms (Kansas City Cardiomyopathy Questionnaire (KCCQ), Functional Assessment of Chronic Illness Therapy-Palliative 14, Hospital Anxiety and Depression Scale (HADS)) and dyadic health (PROMIS-SF Global Health). We have calculated baseline descriptive statistics. Future work includes latent growth mixture modeling to identify distinct symptom trajectories and univariate associations with patient level factors. RESULTS/ANTICIPATED RESULTS: Of 415 patient participants, mean age was 64, 53% were male; 55% were African American; 26% were rural dwellers; 46% had +15.8) and low anxiety (6.7+3.6) and depressive symptoms (5.7+4.3) on the HADS. Of 159 family caregivers participants, the mean age was 57.9, 85.4% were female, 51.9% were African-American, and 65.2% were the patient’s spouse/partner. DISCUSSION/SIGNIFICANCE OF IMPACT: Limited data describes HF symptom pattern trajectories.How co-occurring symptoms affect quality of life or are affected by personal or situational factors are not well-understood. This study will help to identify factors and symptom phenotypes that may serve as targets for future interventions.

